# Articulatory motor planning and timbral idiosyncrasies as underlying mechanisms of instrument-specific absolute pitch in expert musicians

**DOI:** 10.1371/journal.pone.0247136

**Published:** 2021-02-19

**Authors:** Niels Chr. Hansen, Lindsey Reymore

**Affiliations:** 1 Aarhus Institute of Advanced Studies, Aarhus University, Aarhus, Denmark; 2 Center for Music in the Brain, Aarhus University & Royal Academy of Music Aarhus-Aalborg, Aarhus, Denmark; 3 Schulich School of Music, McGill University, Montreal, Quebec, Canada; University of Zurich, SWITZERLAND

## Abstract

The study of musical expertise illustrates how intense training in a specialized domain may instigate development of implicit skills. While absolute pitch, or the ability to identify musical pitches without external reference, is rare even in professional musicians and is understood to have a genetic component, anecdotal evidence and pilot data suggest that some musicians without traditional absolute pitch are nonetheless better able to name notes played on their musical instrument of expertise than notes played on less familiar instruments. We have previously termed this particular gain in absolute pitch identification ability “instrument-specific absolute pitch” (ISAP) and have proposed that this skill is related to learned instrument type-specific timbral and intonational idiosyncrasies and articulatory motor planning activated by the timbre of the instrument. In this Registered Report Protocol, we describe two experiments designed to investigate ISAP in professional oboists. Experiment 1 tests for ISAP ability by comparing oboists’ pitch identification accuracies for notes played on the oboe and on the piano. A subset of the participants from Experiment 1 who demonstrate this ability will be recruited for Experiment 2; the purpose of Experiment 2 is to test hypotheses concerning a mechanistic explanation for ISAP. The outcome of these experiments may provide support for the theory that some individuals have ISAP and that the underlying mechanisms of this ability may rely on the perception of subtle timbral/intonational idiosyncrasies and on articulatory motor planning developed through intensive long-term training. In general, this work will contribute to the understanding of specialized expertise, specifically of implicit abilities and biases that are not addressed directly in training, but that may yet develop through practice of a related skill set.

## Introduction

Expertise constitutes an important topic of study germane to fields such as psychology, cognitive science, and artificial intelligence, where the effects associated with intense, long-term training have been investigated in relation to physiological adaptations and complex cognitive mechanisms [1]. While expertise is often studied in relation to explicit decision-making, as in chess, sports, and military strategy [2], expert performance nearly always entails implicit competences as well [3]. Music is especially informative in studying implicit skill acquisition because of the large variation in experience levels among the general population and because professional musicians undergo intensive periods of training [4] associated with notable implicit stylistic enculturation [5].

Absolute pitch (AP), or the ability to identify musical pitches without external reference to another known pitch, is thought to arise from an interaction of innate and experiential factors [6]. This ability is generally difficult to acquire through explicit training, particularly after an apparent critical period during childhood (however, see [7]) and is also rare even in professional musicians [8–9]. Some AP possessors may demonstrate variance in the strength of their abilities in relation to the harmonic complexity of a note and/or its timbre (e.g. [10–11]). Nevertheless, AP is often associated with individuals who are able to identify pitches with high accuracy across a range of timbres; we refer to this type of AP as “global” AP. Yet, anecdotal evidence and pilot data suggest that some expert musicians who do not possess “global” absolute pitch as such are nonetheless better able to name notes played on their primary musical instrument of expertise than notes played on other, less familiar instruments. Such an ability would suggest that intensive long-term training may lead to the development of a specific variety of absolute pitch in at least some musicians.

In a previous paper [12], we referred to this gain in absolute pitch identification ability for one’s own instrument as “instrument-specific absolute pitch” (ISAP) and proposed a theory of the underlying mechanisms, which we suggest are developed implicitly in at least some musicians during long-term training. Those musicians in our theorized category of “ISAP possessors” would demonstrate a significantly higher pitch identification accuracy for notes played on their primary instrument of expertise as compared to notes played on other instruments. That is, we would expect oboists with ISAP to be able to identify oboe tones more accurately than flute or piano tones, and we would expect flautists with ISAP to be able to identify flute tones more accurately than oboe or piano tones. We have proposed that instrument-specific absolute pitch, which we theorize develops from expert-level familiarity with a musical instrument’s timbre, may use mechanisms distinct from global absolute pitch as it has traditionally been considered.

It should be noted that we do not have reason to believe that global AP and ISAP are mutually exclusive; a given individual may display characteristics of neither ability, one, or both. Specifically, we have operationalized ISAP as the extent to which pitch-labeling performance for one’s primary instrument exceeds performance for other, less familiar instruments—the difference in performance between instruments defines ISAP. Consider three subgroups of people with varying levels of global AP: the first scores at chance level across timbres (“non-AP”), another subgroup scores above chance but below ~85% across timbres (“quasi-AP”), and yet another subgroup scores above ~85% across timbres (“AP”). (For discussion of “quasi” or “partial” AP, see [13–14]). Members of each of these subgroups could have an added advantage for the timbre of one or more of their main instruments, and this added advantage is what we would refer to as “ISAP”—for quasi-AP or AP possessors, ISAP is thus an instrument-specific *gain* in absolute pitch ability. Note, however, that ISAP would offer no additional advantage and thus be impossible to detect in global AP possessors whose accuracy is near or at 100%, as there would be a ceiling effect.

Only a few previous studies have tested the possibility that there may be a pitch-naming advantage for one’s primary instrument in individuals who do not possess strong global AP. In violinists and pianists without global AP, Wong and Wong [15] found that instrumentalists were better able to identify pitches played on their own instruments as compared to sine tones; however, instrumentalists were not tested on non-primary instruments for comparison, and thus the difference in accuracy could plausibly be related to the harmonic complexity of the tones, rather than the familiarity of the timbre. Marvin and Brinkman [16] asked violinists and pianists without global AP to identify synthesized violin and piano tones. While pianists showed an advantage for piano over violin tones, the overall performance of the violinists was not significantly different between the two timbres. Although the authors did not make this proposal, one could imagine this possible difference in ISAP between violinists and pianists could be due to the fact that general aural skills are often practiced with piano timbre, and many non-pianists are expected to acquire basic piano skills. In a group of 12 musicians without global AP who were familiar with the piano as a primary or secondary instrument, Schlemmer, Kulke, Kuchinke, and Van Der Meer [17] only found a pitch-naming advantage for white key notes played on the piano.

Finally, Li [18] did not observe a pitch identification advantage for string timbres in string majors; however, while Li’s participants were not asked to self-identify as either having or not having global AP, they were mostly musicians with very high degrees of global AP ability (as determined in a previous experiment) who began musical training early. We propose that individuals with global AP typically use a different set of mechanisms for absolute pitch identification than do individuals with ISAP. Thus, we do not expect that most global AP possessors will demonstrate use of the mechanisms we propose for ISAP, and accordingly, we would not necessarily expect a similar level of primary-over-other-instrument advantage from these individuals.

Each of these studies has pursued a group-level advantage for primary instrument timbre; however, based on anecdotal evidence and pilot results [12], we suggest that as with global AP, it is likely the case that not all musicians have ISAP or may at least have different degrees of ISAP. In this scenario, research would need to first identify individuals with ISAP. This approach contrasts with the conventional group-level approach of testing for the ability, which assumes that most or all musicians will exhibit an advantage for their primary instrument timbre.

Our theory of ISAP proposes that timbral cues and motor imagery contribute to increased pitch identification accuracy for musical instruments for which one has substantial prior motor and/or timbral experience [12]. This would, for example, be the case for a musician’s primary instrument of expertise.

Our first proposed mechanism entails that musicians with ISAP use timbral cues specific to the type of instrument that they play (oboe, clarinet, etc.) to aid in pitch identification. Such cues may be available via two routes. On one hand, evidence from neuroimaging studies suggests that extreme familiarity with one’s primary instrument has a marked impact on auditory processing [19–21]. Thus, increased or better coordinated cortical processing of the sound of a musician’s primary instrument may facilitate timbre-selective absolute pitch. On the other hand, learned differences in timbre and intonation tendencies among the notes afforded by an instrument may provide clues to pitch identification for the highly experienced instrumentalist.

We have suggested that these potentially useful idiosyncrasies in intonation and timbre can be related to three sources of variation in an instrument’s sound. First, due to continuous physical differences in the elements of tone production (such as length and thickness of strings and air columns), timbre may vary continuously as pitch increases on a given instrument. Second, instruments may contain categorical timbre variations among different registers (such as the chalumeau, throat, clarion, and extreme or “altissimo” registers on the clarinet; see [22]). Third and finally, timbral and intonational idiosyncrasies for specific pitches may be unique to instrument types (e.g. oboe vs. clarinet, [23]); for example, E5 commonly tends to be sharp in intonation on the oboe, but this is not the case across all instruments. Fitzgerald and Ramsey [23], moreover, demonstrated that B♭4 and C5 belonging to the same register on the oboe show notably different overtone spectra with maxima for the third and sixth harmonics, respectively. Finally, while Snow [24] has catalogued common intonational idiosyncrasies among a number of instruments, the full extent of such differences has not been thoroughly studied. However, musicians become intimately familiar with the specificities of their own instrument type during training and practice.

Our previous paper [12] proposed the three types of timbre and intonation tendencies that may relate to ISAP, described above, and suggested that the prominence of ISAP among players of a certain type of instrument may be related to the extent to which that instrument displays such tendencies. Given the lack of intonational idiosyncrasies on a modern, equally-tempered piano and the more homogenous modes of tone production across the registral range of both pianos and, to a certain extent, string instruments like the violin, there is some reason to believe that both idiosyncratic timbre and intonation may be relatively more prominent on a double-reed, woodwind instrument like the oboe. Yet, specialized practitioners of such instruments have not received the same attention as keyboard and string players in previous research on absolute pitch. We expect that such idiosyncrasies will translate to different extents among different instruments. Thus, our choice to begin the investigation of ISAP with oboe players is based on the observation that the oboe has a relatively high number of intonational and timbral idiosyncrasies as well as relatively fixed mappings between motor patterns and absolute pitches.

Our second proposed mechanism of ISAP entails that articulatory motor imagery, as stimulated by the experience of hearing one’s primary instrument, facilitates absolute pitch identification. Pitch identification accuracy with one’s primary instrument timbre may be due in part to learned connections between sounds and the kinesthetic actions required to produce those sounds [25]. Related bisensory correspondences between absolute pitch categories and visual notation have been demonstrated in AP possessors [26], and internalized motor representations have been related to the widespread ability to identify and produce music at absolute tempo without external reference [27]. In musical practice and performance, musicians constantly connect their motor actions with the sound that is produced [28–29]. When a musician hears a note played on their primary instrument, motor areas of their brain that are involved in producing that sound are activated; for example, Furukawa, Uehara, and Furuya [30] found that expert pianists demonstrated muscle-specific M1 excitability in response to listening to synthesized piano tones while non-musicians did not (see also [31–32]). We propose that this kinesthetic memory aids the musician in pitch identification. In the case of wind players, for example, this kinesthetic memory is likely related not only to hand and finger position, but also to embouchure and the tongue, lips, and jaw. Indeed, Choi et al. [33] observed differences in cortical thickness in areas related to the lips and tongue in wind instrumentalists as compared to non-musician controls.

In the two case studies reported by Reymore and Hansen [12], we tested for increased accuracy in pitch identification for oboe tones over piano tones in professional oboists. Of the two oboists tested, one demonstrated ISAP; this oboist’s performance provided evidence consistent with the hypotheses that ISAP ability relied on articulatory motor planning and timbral cues. These timbral cues appeared to be driven by pitch-specific idiosyncrasies of the oboe as an instrument type rather than by familiarity with a specific instrument exemplar (i.e., the player’s personally-owned instrument) or by familiarity with one’s own tone quality. Although the lack of initial supporting evidence for an advantage for one’s personal instrument exemplar or style of playing does not disprove that such effects may indeed exist, they are not tested here (see Reymore & Hansen [12], for further commentary).

In this Registered Report Protocol, we outline the first full-scale formal test of the newly proposed theory of instrument-specific absolute pitch ability. In the subsequent Registered Report Research Article, we will report the results of two experiments with a sample of professional oboists. Experiment 1 tests for the presence and prevalence of ISAP. Having identified individual oboists with ISAP, the purpose of Experiment 2 is to test two proposed mechanisms for ISAP, instrument type-specific timbral/intonational cues and articulatory motor planning, in the sub-group of oboists who demonstrate ISAP in Experiment 1.

## Methodology

All experiments involving human participants will be conducted with approval from the Institutional Review Board of Danish Neuroscience Centre at Aarhus University/Aarhus University Hospital (DNC-IRB-2020-001). This approval was obtained on 30th June 2020 and is valid until 30th June 2022. Informed consent will be obtained by checking a box on the online platform, and participants will verbally verify to the experimenter that they are ready to move on before proceeding.

### Experiment 1

The goal of Experiment 1 is to identify individuals who exhibit instrument-specific absolute pitch (ISAP). Consequently, the hypothesis below will be tested for each participant.

#### Hypothesis

For each individual oboist, we hypothesize that they will demonstrate ISAP, which we define as (1) identifying pitches played on the oboe with an accuracy that is above chance level, assuming octave equivalence (i.e., 1/12 = 8.3%), and (2) doing so with an accuracy that is significantly higher for oboe tones than for piano tones.

#### Experimental design & statistical analysis

In this experiment, participants will listen to oboe and piano tones and identify the name of each pitch played from a set of all 32 possible pitches. An experimental design with instrument as a two-level, within-participant factor (oboe vs. piano) will be used. Further information on the choice of piano as a relevant contrast timbre is given below in the section “Pilot study to determine suitable contrast timbre for Experiment 1.”

Data from Experiment 1 will be analyzed at the subject level using the statistical procedure from a previous pilot experiment [12]. Specifically, the first criterion for ISAP, above-chance performance for oboe tones (i.e., accuracy > 8.3%), will be assessed with a one-sample proportions test with continuity correction. We will assess the second criterion of significantly better performance for oboe tones over piano tones with Pearson’s chi-squared tests with Yates’ continuity correction.

Moreover, degrees of variance around the correct pitch value will be assessed by applying Mann-Whitney *U*-tests to the absolute number of semitones that incorrect responses are off from the target pitch (i.e., “absolute semitone errors”). While the criteria for participant inclusion in Experiment 2 are not related to absolute semitone errors, tests of variance around the correct pitch will be used for illustrative purposes and may be related to individual factors in exploratory *post hoc* analysis, including response times, number of replays, musical experience, and recent practice habits. As suggested by a reviewer, we will also explore whether there tends to be greater confusion among pitches that are harmonically related, namely octaves, fifths, and thirds.

Finally, we will perform exploratory analysis to determine whether there are differences between responses of participants with ISAP who also demonstrate some degree of AP ability (i.e., identify more oboe than piano tones and identify piano tones above chance) and those who do not demonstrate any degree of AP ability (i.e., identify more oboe than piano tones but identify piano tones at or below chance). We will also assess to what extent ISAP and evidence of above-chance AP tend to co-occur in our sample.

#### Pilot study to determine suitable contrast timbre

A follow-up pilot experiment was first conducted on Oboist 1 from the previous pilot study (i.e., the second author of this report) with three goals: first, we wanted to establish if her ISAP skill could be replicated at a later date (about 17 months after the first pilot study) as evidence of stable performance and good test-retest reliability. Second, we wanted to determine if her ISAP skill could be replicated with alternative stimuli from a widely used sound bank with tones that were not recorded by herself or on her own instrument. Third and finally, we wanted to test empirically if ISAP accuracy would be even lower for an alternative contrast timbre such as flute or violin with which she was less familiar than with piano. The final goal was motivated by the concern that since piano is used widely in theory and aural skills classes and since many musicians—including Oboist 1—are expected to acquire basic piano skills, piano timbre and the physiological idiomatics of playing the piano could be sufficiently more familiar to Oboist 1 than the timbres and idiomatics of other instruments, and thus this familiarity might affect pitch-naming accuracy. Therefore, it was predicted that the pitch identification skills of oboists with ISAP might be even lower for the flute and violin. Note that while the use of sine tones would in theory eliminate the confound introduced by using instruments with which participants potentially have varying levels of experience, we would be unable to determine whether improved performance for oboe tones was a result of increased instrument-specific familiarity or of the overall difference between simple and complex tones. Specifically, in global AP possessors, accuracy of pitch identification decreases from natural complex tones to pure sine tones (e.g., [34–38]).

To test empirically whether Oboist 1 would have lower pitch labelling accuracy with an alternative contrast timbre (other than piano), oboe, piano, violin, and flute stimuli were taken from the *McGill University Master Samples* (MUMS) [39]. Eerola and Ferrer [40] report finding a number of labeling errors and intonational inaccuracies in the MUMS library. In light of this criticism, before proceeding to conduct this pilot study, we carefully confirmed that none of the specific MUMS files used here suffered from these issues.

This pilot experiment comprised eight blocks, each containing 15 tones played on a single instrument, that were presented to the second author in a reverse counterbalanced order of oboe-flute-piano-violin-violin-piano-flute-oboe. Across the two blocks for each instrument, a total of 30 pitches ranging from C4 to F6 (in Scientific Pitch Notation) were presented for each instrument. Because not all pitch levels from our previous study [12] were included in the MUMS library and/or could be played on all instruments, this range represents a slight reduction from the conventional range of the oboe.

Based on the results of this follow-up pilot study, Oboist 1 continued to meet both criteria for ISAP. Specifically, she identified four out of every five oboe tones on average, which is comparable with (or rather exceeds) her 66.2% correct performance as reported for both sub-studies of our previous pilot study [12]. One-sample proportions tests with continuity correction confirmed that she indeed identified oboe tones more accurately than the predetermined chance level of 8.3% (80.0% [95% CI: 60.9–91.6%], *χ*^*2*^(1) = 192.44, *p* < .0001), and Pearson’s chi-squared tests with Yates’ continuity correction further showed that her performance for oboe tones was significantly more accurate than for piano tones (23.3%, *χ*^*2*^(1) = 17.09, *p* < .0001), flute tones (23.3%, *χ*^*2*^(1) = 17.09, *p* < .0001), and violin tones (40.0%, *χ*^*2*^(1) = 8.40, *p* < .0037). These results provide evidence of stable performance over time, good test-retest reliability, and reliable performance with a new set of sound stimuli. As in the previous pilot study, percentage accuracy reported here is reported under the assumption of octave equivalence. However, only a single octave confusion (where the correct pitch class was identified in the wrong octave) appeared across the entire dataset (on the flute).

In sum, despite Oboist 1’s experience with piano as a secondary instrument, identification for piano was not more accurate than for either flute or violin, instruments with which she has had no experience playing. As a result, we decided to continue using piano stimuli as a contrast timbre in Experiment 1. Although Oboist 1’s accuracy in labeling flute pitches was equal to piano pitches, piano pitches provide an advantage in that they have been more commonly used in previous studies of absolute pitch, and thus we will be able to compare our results more directly with previous literature.

#### Stimuli

Based on the success with this soundbank in the timbre pilot experiment described above, oboe and piano stimuli were taken from the *McGill University Master Samples* (MUMS) [39]. Both types of stimuli spanned the full range of the 32 available chromatic pitches from the standard range of the oboe from B♭3 to F6. Oboe tones were played at a medium dynamic level with moderate amounts of vibrato ranging from 2 to 3 seconds in duration. Piano tones were sourced from the “loud” subsample of piano tones with approximately 3 seconds duration played on a 9’ Steinway Model D Concert Grand Piano produced at the factory in Hamburg.

Stimulus preparation and editing for the current experiments was carried out in Cubase 7.0.5. Recorded tracks were segmented into single-tone clips using the Split Function starting at 1 second before tone onset with a total clip duration of 5 seconds. All clips were peak-normalized to +18 dB FS (with no pre- or post-crossfading) and exported as MPEG 1 Layer 3 (“mp3”) files in stereo with 160 kbps bit rate and 44.1 kHz sample rate. The pitch of each single-tone clip was confirmed using the Android app “Vocal Pitch Monitor” created by Tadao Yamaoka.

#### Simulations

A suitable number of trials for Experiment 1 was estimated via simulations in R [41] adopting the previously described statistical procedures and a predetermined, conventional alpha level of .05. Data for these simulations were randomly drawn from the case-study data from our previously published pilot experiment [12]. In that pilot experiment, we tested two professional oboists (including the second author of the current paper) on the tasks described in this protocol with 68 oboe trials and 68 piano trials, which took approximately two hours to complete for each oboist. Because this duration is impractical to implement for a larger group of oboists (especially in combination with Experiment 2), the purpose of the simulations reported here was to determine an appropriate compromise between statistical power and experiment length. Because only Oboist 1 and not Oboist 2 showed significant ISAP in the pilot experiment, simulation data to determine the number of trials needed to establish ISAP (when it is present) were exclusively sampled from Oboist 1 (the second author of the current paper).

Note that our definition of ISAP is solely concerned with the difference in performance between primary instrument (oboe) tones and a contrast timbre (piano). However, we are also interested in whether low absolute semitone error values are characteristic of some or perhaps all musicians with ISAP, and so we included both factors in our simulations.

**Fig 1:**
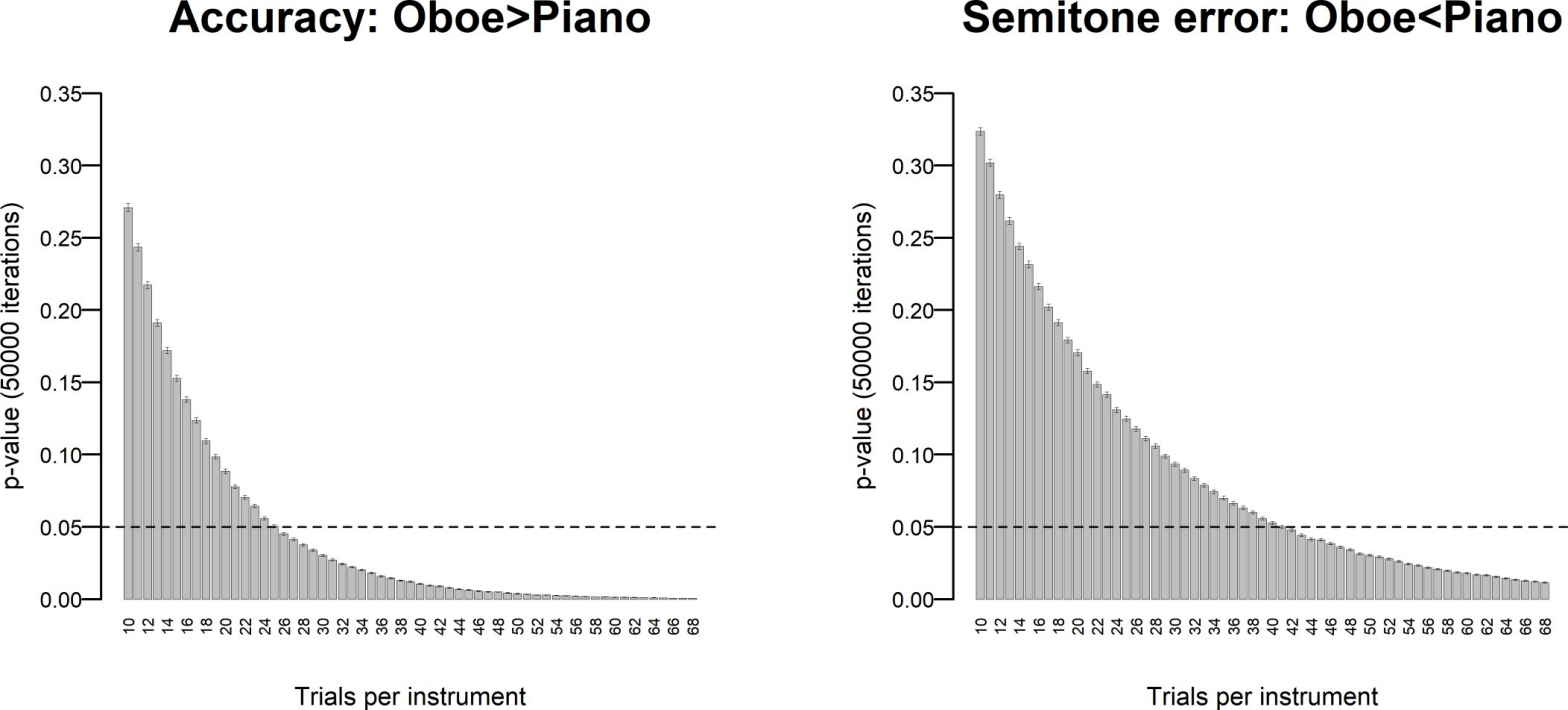
Simulations, Exp 1. Simulations on random samples of pilot data from Experiment 1 with number of trials per instrument (oboe and piano) on the x-axis and mean *p*-value (across 50,000 iterations) on the y-axis. The left panel depicts accuracy whereas the right panel depicts absolute semitone error values for incorrect responses. Error bars indicate 95% confidence intervals.

Simulations with 50,000 iterations were conducted in the following way: For each possible sample size ranging from 10 to 68 trials per instrument (i.e., piano and oboe), 50,000 subsets (comprising the relevant number of trials) were randomly drawn from Oboist 1’s empirical data. For each subset, the difference in pitch-identification performance between oboe and piano was then assessed in terms of accuracy using a Pearson’s chi-squared test with Yates’ continuity correction and in terms of absolute semitone error using a Mann-Whitney *U*-test. This resulted in 2,950,000 unique *p*-values for accuracy and 2,950,000 unique *p*-values for absolute semitone errors. Mean *p*-values with 95% confidence intervals were finally computed for each sample size of trials (Fig 1).

These simulations showed that we can detect the same size of ISAP effect as shown by Oboist 1 in our pilot experiment with 95% confidence and an alpha level of .05 if we use at least 26 trials per instrument (oboe and piano) in terms of accuracy and at least 42 trials per instrument in terms of absolute semitone error values. Oboist 1’s accuracy values correspond to an odds ratio (an unstandardized effect size statistic) of 2.65 for accuracy (resulting from 66.2% accuracy for oboe tones and 25.0% accuracy for piano tones, cf. [12]). For absolute semitone errors, the effect size was *r* = .382 (cf. [12]). Since our ISAP criteria rely on accuracy rather than semitone error values, and because the full range of the oboe samples used here contains 32 pitches from B♭3 to F6, we deemed that 32 trials per instrument would be sufficient, thus reducing the duration of Experiment 1 to a practically feasible ~60 mins.

**Fig 2:**
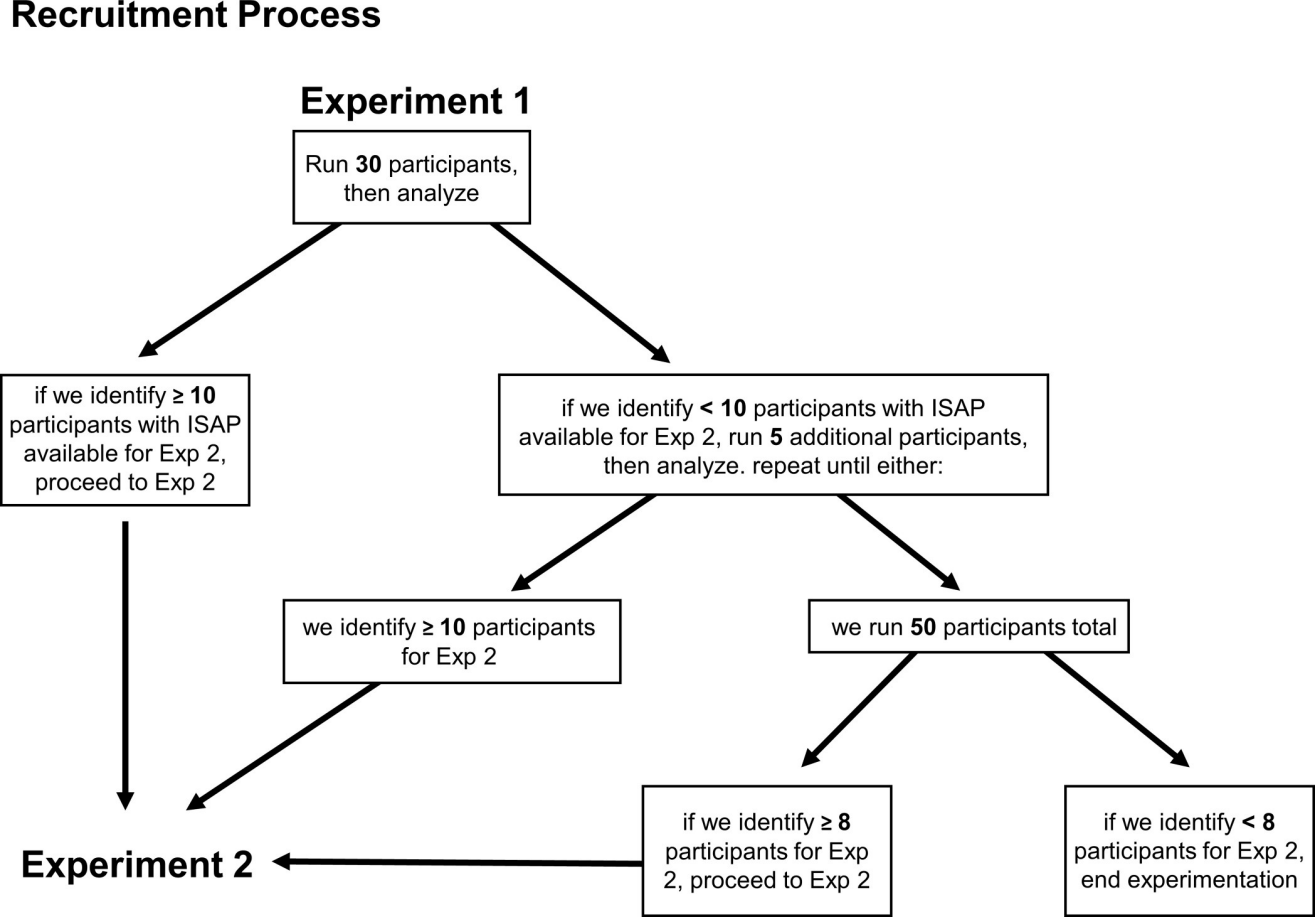
Recruitment process. Participants for Experiment 2 will be recruited from the subset of participants in Experiment 1 who demonstrate ISAP. Our simulations suggest that we will need at least eight participants in Experiment 2 in order to observe whether transposition and motor tasks interfere with ISAP. However, as the prevalence of ISAP among oboists is unknown, we designed the recruitment process for Experiment 1 to allow for the possibility that we will need to recruit more than the initial goal of 30 participants in order to identify a sufficient number of participants for Experiment 2. In sum, 30–50 oboists will participate in Experiment 1, and running Experiment 2 requires a minimum of eight participants but may include more.

Given that the purpose of Experiment 1 was to screen participants for ISAP ability (i.e., compliance with the inclusion criteria for Experiment 2), the final number of participants required for Experiment 1 can only be determined once we have estimated the required sample size for Experiment 2. The recruitment process and the relationship between participant numbers in Experiments 1 and 2 is illustrated in Fig 2; for further detail, see the “Simulations” section of “Experiment 2” below.

#### Participants

Based on the simulations for Experiment 2 described later and our empirical estimates of the prevalence of ISAP, we estimate that a minimum of 8 participants and a maximum of 50 participants may be needed for Experiment 1 in order to be able to obtain sufficient participant numbers for Experiment B. This assumes a zero dropout rate between the two experiments, which may be somewhat optimistic. However, this will likely be compensated for by our conservative estimate of the prevalence of ISAP. Specifically, one of the two oboists tested in the pilot experiment demonstrated ISAP [12]. For the purpose of determining participant numbers for Experiment 1, we estimated that ISAP may be 2.5 times less prevalent than this empirically derived frequency of 50% suggests; that is, our conservative estimate of the prevalence of ISAP for the purposes of determining minimum sample size is 20%.

Initially, we determined that in order to go ahead with group-level analysis in Experiment 2, we would need a minimum of 8 oboists with ISAP (see Fig 6 below for further explanation); ideally, however, we would recruit a larger group of participants, which would increase the power of the analyses (and allow for the possibility that participants with significant ISAP will show weaker levels of ISAP than Oboist 1 in our previous experiment). This would be particularly important if Oboist 1, who provided the data for the simulation, has above-average ISAP ability as found amongst ISAP possessors.

Consequently, we devised the following procedure for recruitment in Experiment 1 with the goal of recruiting at least 10 participants for Experiment 2, two more than the minimum suggested by the simulations. We will set an initial recruitment goal of 30 participants for Experiment 1. After we run 30 participants, we will examine the data to determine how many of the participants demonstrate evidence of ISAP ability. If we have at least 10 participants who both demonstrate ISAP ability and subsequently agree to participate in Experiment 2, we will conclude data collection for Experiment 1. However, if we do not identify 10 potential participants for Experiment 2 after collecting data from 30 oboists in Experiment 1, we will continue recruitment up to a maximum of 50 oboists. The additional recruitment will occur in groups of five; that is, we will examine the new data (on the individual level) after running five participants. If this new data reveals that we have identified a total of 10 potential participants for Experiment 2, we will stop data collection for Experiment 1; if we have not yet identified 10 potential participants, we will recruit another five oboists. This process will repeat up to a maximum of 50 participants for Experiment 1. Crucially, because analysis of data from Experiment 1 is conducted on the individual level (rather than on the group level), the described process will not lead to multiple testing on the same data. Once we recruit our *a priori* maximum of 50 participants, we will proceed with Experiment 2 only if we are able to identify at least 8 participants with ISAP (again, ideally we will have identified 10 or more, but 8 represents the minimum number of participants needed in Experiment 2 according to the simulations reported below). This *a priori* cap of 50 oboists was determined in regard to the practicality of data collection, the relatively small pool of eligible participants (i.e., the global population of professional oboists), and the estimation that ISAP may be up to 2.5 times less prevalent than the rate of 50% suggested by the pilot case study (see above).

To this end, professional oboists will be recruited via social media, email listservs, and individual emails to oboists known to the authors to be eligible for the experiment, which will be advertised as “a music cognition study about pitch perception.” To qualify for participation in the experiment, potential participants must be over 18 years old, self-identify as either current professional or current pre-professional oboists, and self-report at least five years of regular experience playing the oboe.

#### Procedure

To avoid carryover effects between oboe and piano tones—where superior performance for one instrument could be used to guess the correct pitch of tones played on the other instrument via relative pitch kept in working memory—the stimuli will be presented in four blocks, two containing only oboe tones and two containing only piano tones, presented in a reverse counterbalanced order. The use of either oboe-piano-piano-oboe or piano-oboe-oboe-piano is further counterbalanced across participants. All blocks will be presented using the Gorilla Experiment Builder (www.gorilla.sc) [42].

**Fig 3:**
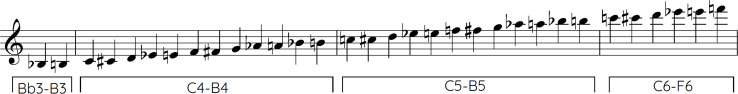
Range of stimuli. Diagram depicting the range of pitches from B♭3 to F6 included in the current study. This corresponds to the standard range of the oboe (while the oboe can play higher than F6, occurrences of these higher pitches are extremely rare in most musics).

Participants will meet with one of the experimenters via Zoom video conferencing software. After verifying that participants have headphones and will use them throughout the experiment, and that participants are in a quiet place without distractions or interference from background noise, the experimenter will read the instructions out loud while the participant follows along on screen. During these instructions, the experimenter will describe the system of scientific pitch notation and provide a diagram (shown below in Fig 3), answering any questions the participant may have about this pitch labeling system and confirming that they understand. The diagram will be visible to participants throughout the experiment, and they will be encouraged to refer to it as needed.

Next, we will collect information related to demographics and musical background. Demographic questions will relate to age and gender. Because the predicted mechanisms are derived from experience, it may also be possible that recent activity playing an instrument may have some effect on the strength of ISAP demonstrated. While this is not a variable we are able to manipulate within the practical constraints of this experiment, we plan to collect information on participants’ practice habits. For both piano and oboe, participants will be asked how many years ago they began playing, the total number of years they have regularly played, how many hours they have played (practice or performing) within the last week, and about how many hours they played per week on average throughout the last six months. Participants will be asked to self-identify using a single-question measure of musical sophistication ([43], see also [44]) and to report the age at which they began musical training. Finally, information about solfege training will be collected, including age of commencement, type (moveable or fixed), and whether the participant currently uses any particular solfege system. Participants will also be asked to self-report whether they consider themselves to have absolute pitch (with the response options “yes, definitely,” “maybe/to some extent,” and “no, definitely not”) and to estimate the percent of pitches that they think they will be able to identify in an absolute pitch identification task (note that we will not mention possible effects of timbre).

**Fig 4:**
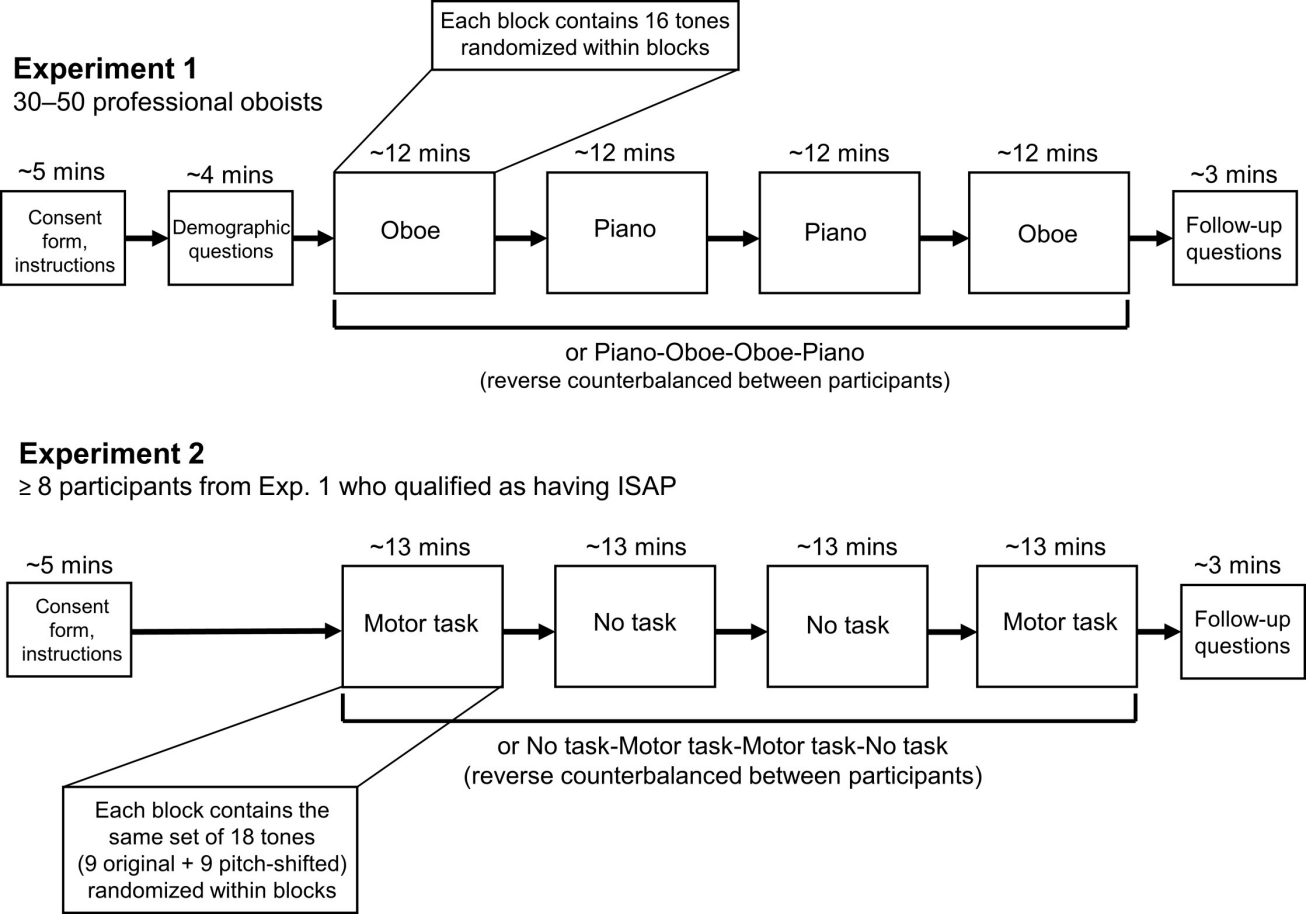
Experimental design. Experimental designs for both Experiment 1 and Experiment 2 are illustrated. Both experiments consist of four principal blocks presented in counterbalanced order, where stimuli within each block are presented randomly.

**Fig 5:**
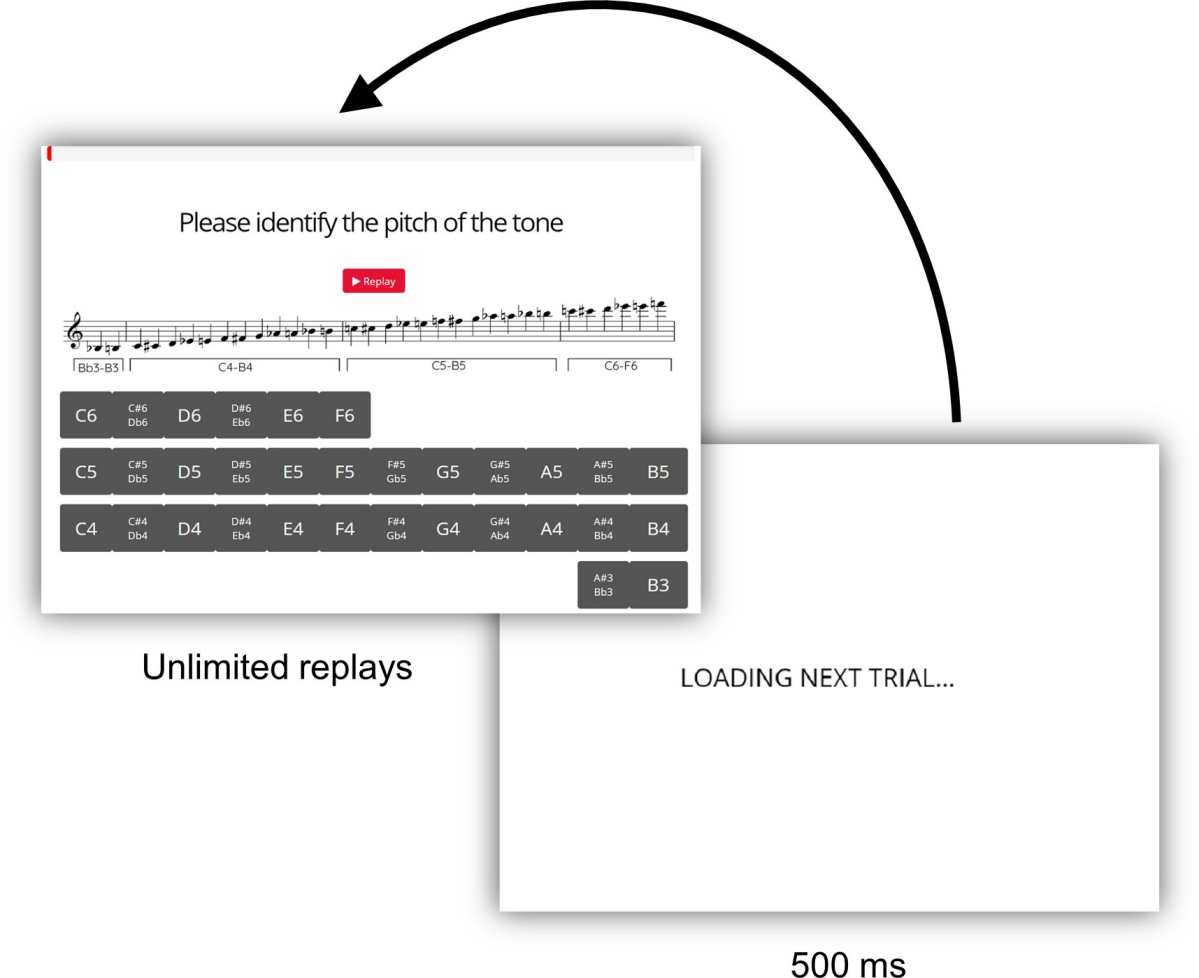
Trial interface. The trial interfaces for both Experiments 1 and 2 are identical; participants will listen to a tone and select the name of the note. Participants may replay tones as many times as they wish. Immediately after they have responded by clicking a button on the screen using the computer mouse, a loading screen will be shown for 500ms to avoid duplicate responses to the same trial.

The 32 tones for each instrument will be randomly distributed between the two blocks for each instrument, and the 16 tones within each of the four blocks will be presented in random order (see Fig 4). Oboists will choose each pitch name from a set of all 32 possible pitches with both enharmonic equivalents listed when appropriate (e.g., F#4/G♭4; see Fig 5 for screen layout). Along with each question, a diagram will be displayed showing the correspondences between scientific pitch names and notes on a staff in treble clef (Fig 3). During the experiment, further quantitative data will be collected in terms of response times and number of replays.

After the experiment, participants will be prompted to (a) provide a free-form response to describe any specific response strategies they may have used (if any) and (b) rate on a 7-point Likert-scale how confident they felt in their responses overall (anchored from “very unconfident” to “very confident”). The concept of ISAP will be briefly explained, and participants will then be asked whether they believe they have this ability, and whether the ISAP phenomenon was something that they were aware of before taking part in the study.

Note that it remains possible that a participant may have a particular pitch memorized (for example, the tuning ‘A’) and is able to use this memorized pitch as a reference tone, along with their relative pitch, to infer the names of other pitches. If the pitch itself is memorized, this strategy would likely work equally well with any timbre. On the other hand, it is possible that recognition of a particular pitch and subsequent use of relative pitch may be facilitated by the timbre of the primary instrument: for example, an oboist may be more likely to recognize the oboe note A4, or a string player might be more likely to recognize the pitches of their open strings. Musicians using such methods would demonstrate improved pitch-recognition for the timbre of their primary instrument but would be using a mix of absolute and relative pitch strategies. In the current study, such a musician would be classified as having ISAP. Importantly, it is impossible to use a reference tone in the experimental scenario without some type of absolute pitch, even if it is for a single note, and thus their absolute pitch ability for a particular note from their primary instrument is resulting in an advantage for pitch-labeling for their primary instrument timbre. Moreover, use of relative pitch may show up as no interference with accuracy from the pitch-shifting manipulations employed in Experiment 2 as described below. Post-experiment questions targeting individual differences in response strategies will help to clarify whether mixed use of absolute and relative pitch is a common strategy and should be investigated as a subtype of ISAP in future studies.

### Experiment 2

Experiment 2 is designed to investigate the potential underlying mechanisms of instrument-specific absolute pitch (ISAP). Specifically, we will be testing whether our pilot findings that ISAP identification is subject to deterioration from artificial pitch-shifting and a motor interference task [12] generalize to a wider population of expert oboists with established ISAP ability.

#### Hypotheses

Based on the results from our pilot study [12], it was hypothesized that:

**H1.** Oboists with ISAP will be less successful in identifying the pitch of artificially pitch-shifted oboe tones than the pitch of non-transposed oboe tones.
**H2.** Oboists with ISAP will be less successful in identifying the pitch of oboe tones in the motor interference condition as compared to the condition with no motor interference.

For the purpose of testing these hypotheses, we operationalize “successful” pitch identification in two ways: (a) accuracy (i.e., proportion of correct responses) and (b) precision (i.e., absolute semitone error values).

#### Experimental design & statistical analysis

Experiment 2 consists of the same type of task used in Experiment 1; that is, participants will listen to recordings and respond by selecting the pitch. However, in this Experiment, only oboe tones will be used. To this end, a full factorial design with the two-level factors pitch-shifting and motor interference will be used for Experiment 2.

Data from Experiment 2 will be analyzed at the group level using an extension of the statistical procedures from our individual-level pilot experiment [12]. Mixed-effects logistic regression will be conducted using the *glmer()* function from the *lme4* package [45] in R [41], with pitch-shifting and motor interference as fixed (binary) effects and random intercepts for each participant. Because interaction effects are not hypothesized (and did also not improve the fit in *post hoc* analysis of our pilot data [12]), they will not be modelled here. Further, to model the degree of variation around the correct pitch value, cumulative link models will be fitted to the absolute semitone error values using the *clmm()* function from the *ordinal* package [46] in R with the same fixed and random effects.

The experimental design described here allows for secondary analysis that is not directly motivated by our main hypotheses. First, we will explore whether response times and/or number of replays are related to task success. Second, in the pilot data, we found in secondary analysis that modeling the pitch-shifting as a continuous rather than binary variable improved the model slightly and lowered both the Akaike and Bayesian Information Criteria. This suggests that pitch identification accuracy may decrease as the number of semitones that tones are shifted increases. However, in that specific pitch levels are always shifted by a predetermined number of semitones (to ensure that the overall pitch set is identical for pitch-shifted and non-pitch-shifted tones), the experimental design used both here and in the pilot study [12] does not manipulate degree of pitch-shift independently from absolute pitch. Therefore, proper hypothesis-testing would require a research design optimized for answering this question. Accordingly, in secondary analysis, we will explore potential trends related to pitch-shift distance via regression with pitch-shifting as a continuous rather than binary variable, which we anticipate will suggest whether this variable should be tested in a future experiment.

#### Simulations

**Fig 6:**
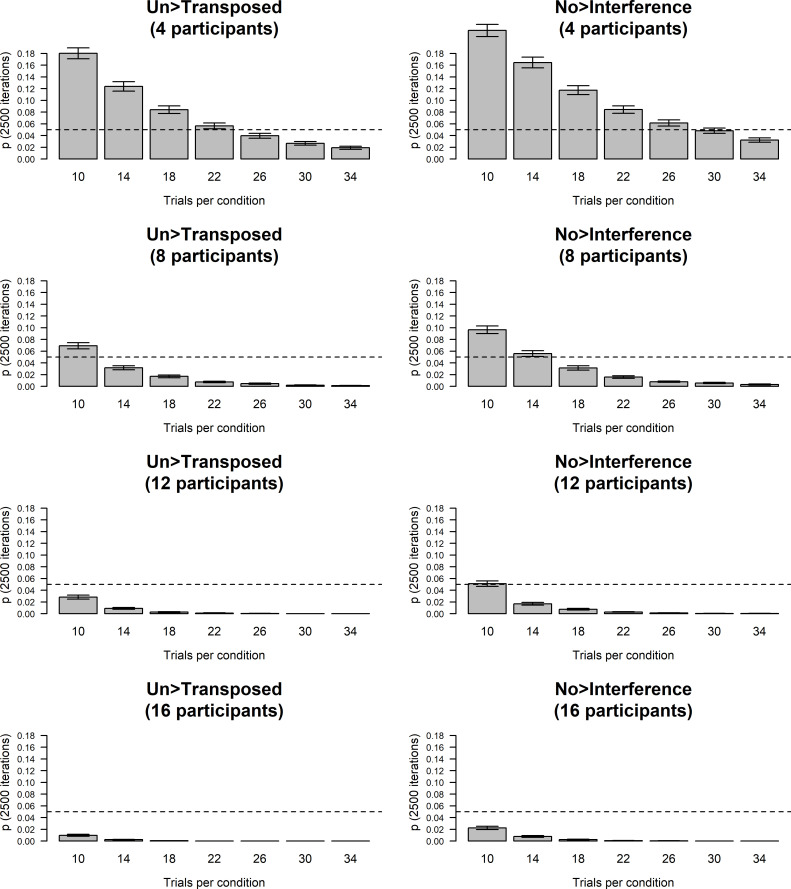
Simulations, Experiment 2, accuracy. Simulations on random samples of accuracy pilot data from Oboist 1 in Experiment 2 with the number of trials in each of the four conditions on the x-axis (pitch-shift vs. original crossed with motor interference vs. no interference) and mean *p*-value (across 2,500 iterations) on the y-axis. The left panel depicts effects of transposition whereas the right panel depicts effects of the motor interference task. Error bars indicate 95% confidence intervals.

**Fig 7:**
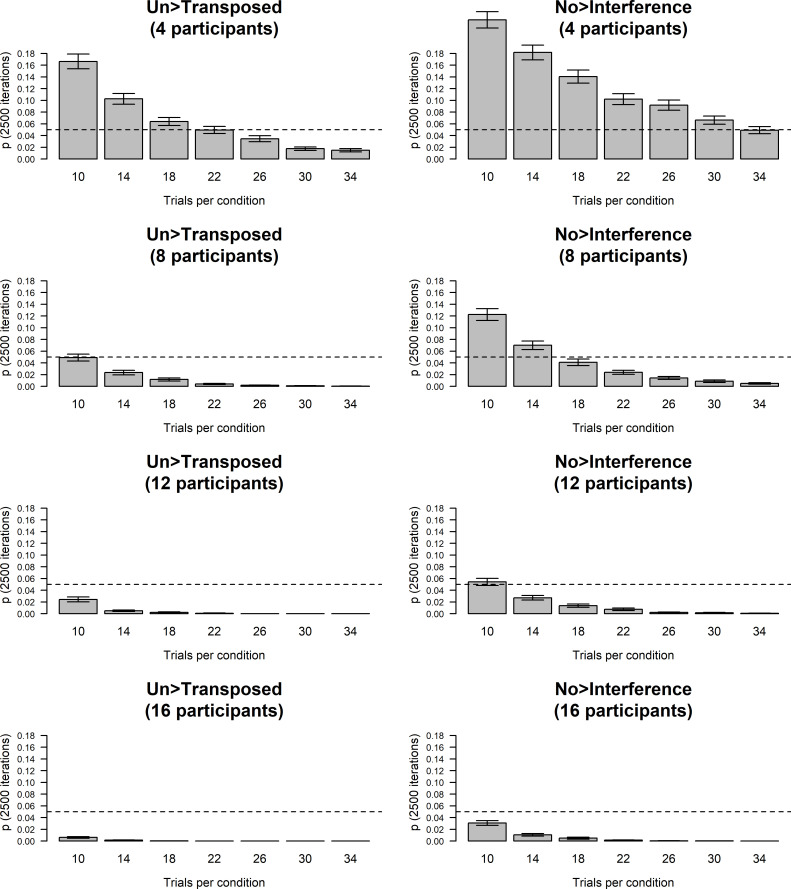
Simulations, Experiment 2, absolute semitone error. Simulations on random samples of absolute semitone error pilot data from Oboist 1 in Experiment 2 with the number of trials in each of the four conditions on the x-axis (pitch-shift vs. original crossed with motor interference vs. no interference) and mean *p*-values (across 2,500 iterations) on the y-axis. The left panel depicts effects of transposition whereas the right panel depicts effects of the motor interference task. Error bars indicate 95% confidence intervals.

Suitable sample sizes and number of trials for Experiment 2 were estimated via simulations in R [41] adopting the statistical procedures above and a predetermined alpha level of .05. As described for Experiment 1 above, data for these simulations were randomly drawn from Oboist 1’s data from our previous pilot experiment [12]. The pilot data for Experiment 2 comprised a full-factorial 2x2x2x2 design with 32 trials in each condition with stimuli played by the participant themselves or the other participant (self vs. other) on their own oboe or the other participant’s oboe (own oboe vs. other oboe) as well as with the pitch-shifting (original vs. pitch-shifted) and motor interference manipulations (motor interference vs. no interference). Ordinal logistic regression analysis on data from Oboist 1 had previously shown significant effects of pitch-shifting (*OR* = 1.81 [95% CI: 1.30–2.53]) and motor interference (*OR* = 1.60 [95% CI: 1.15–2.23]) whereas the effects of performer (*OR* = 0.86 [95% CI: 0.62–1.20]) and instrument (*OR* = 0.97 [95% CI: 0.69–1.35]) were non-significant [12]. Thus, given the two non-significant results demonstrated in the pilot study, the levels of the performer and instrument factors were collapsed for the purpose of the current simulations, resulting in a total of 136 trials in each of the four conditions (2x2 design) planned for Experiment 2 (i.e., untransposed/no interference, transposed/no interference, untransposed/motor interference, transposed/motor interference). Separate simulations with 2,500 iterations were run for virtual datasets with 4, 8, 12, and 16 simulated participants, respectively, following this procedure: For the sample sizes of 10, 14, 18, 22, 26, 30, and 34 trials per each of the four conditions, 2,500 subsets (comprising the relevant number of trials per condition) were randomly drawn from Oboist 1’s empirical data for each of the simulated participants. For each subset, unique *p*-values were obtained for the fixed effects pitch-shifting and motor interference by fitting a mixed-effects logistic regression model to the accuracy data and a cumulative link model to the absolute semitone error data as described under “Experimental design & statistical analysis” above. Mean *p*-values with 95% confidence intervals were finally computed for each sample size of trials per condition and for each sample size of participants (Figs 6 and 7).

These simulations showed that (adopting an alpha level of .05) we can expect to detect significant effects of transposition (Fig 6, left panel) and motor interference (Fig 6, right panel) with 95% confidence if we choose a suitable combination of number of trials per condition and tested participants with ISAP ability as detected in Experiment 1. Similar simulations are available for absolute semitone error values in Fig 7. By visual inspection, we deemed that we would have reasonable power if we recruited at least 8–10 participants with demonstrated ISAP ability and included ~18 trials per condition, reducing the overall duration of Experiment 2 to ~60–70 mins.

Note, however, that the number of participants available for Experiment 2 will depend on the proportion of participants with ISAP identified in Experiment 1 (see Fig 2). At this time, based on our pilot data, our best estimate is that ~50% (i.e., 1 out of 2) of professional oboists will exhibit significant ISAP. This is, however, an extremely uncertain estimate, and we expect that the prominence of ISAP ability could be up to 2.5 times lower (i.e., ~20%) in the general population of professional oboists. This would necessitate that we recruit up to 50 participants for Experiment 1.

#### Stimuli

The oboe tones from the MUMS soundbank [39] used for Experiment 1 will be used again in Experiment 2. Sound clip segmentation, peak-normalization, and file formats are thus as described above.

For the purposes of generating the pitch-shifted stimuli, the Pitch Shift function in Cubase 7.0.5 will be used to manipulate a copy of each sound clip. Specifically, pitch will be shifted up or down according to a pattern by which every consecutive set of eight pitches is transposed by +4, -1, +3, -2, +2, -3, +1, and -4 semitones. This process results in a new set with the same sounding pitches as the original one (see Table 1) and ensures that transpositions differ between consecutive octaves. Pitch shifting will use the Time Correction setting to ensure that the duration of each clip stays the same as well as the Solo Musical setting, which uses a high-quality algorithm optimized for offline processing of monophonic musical material. Formant Preservation will not be applied because this setting generated clearly audible artefacts manifesting as background “whirling” noises before and after the oboe tones when preparing stimuli for a previous experiment [12].

**Table 1:** Template for generating the pitch-shifted oboe stimuli variants used in Experiment B.

Original pitch	Transposition (semitones)	Modified pitch
B♭3	+4	D4
B3	-1	B♭3
C4	+3	E♭4
D♭4	-2	B3
D4	+2	E4
E♭4	-3	C4
E4	+1	F4
F4	-4	D♭4
G♭4	+4	B♭4
G4	-1	G♭4
A♭4	+3	B4
A4	-2	G4
B♭4	+2	C5
B4	-3	A♭4
C5	+1	D♭5
D♭5	-4	A4
D5	+4	G♭5
E♭5	-1	D5
E5	+3	G5
F5	-2	E♭5
G♭5	+2	A♭5
G5	-3	E5
A♭5	+1	A5
A5	-4	F5
B♭5	+4	D6
B5	-1	B♭5
C6	+3	E♭6
D♭6	-2	B5
D6	+2	E6
E♭6	-3	C6
E6	+1	F6
F6	-4	D♭6

#### Participants

As described earlier, the available number of participants for Experiment 2 will depend on the proportion of participants in Experiment 1 who show significant levels of ISAP. The statistical simulations suggest that a minimum number of 8 eligible participants will be required in order to make inferences about the possible detrimental effects of pitch-shifting and motor interference on absolute pitch identification.

#### Procedure

To test for effects of motor imagery on ISAP, we developed an interference task that is expected to impair pitch-naming accuracy because it increases demands on motor-related brain areas involved in playing the instrument. In the case of the oboe, this includes the hands and fingers as well as lips and jaw, which are called upon for crucial embouchure adjustments while playing.

Motor tasks may interfere with auditory imagery; for example, Beaman, Powell, and Rapley [47] found that chewing gum has negative effects on spontaneous musical recollection (earworms), in support of the idea that chewing gum interferes with motor-related subvocalization or subvocalization-like processes that are linked to earworms. Consequently, a motor interference task will be implemented in Experiment 2. Although this gum-chewing task was used in the pilot experiment, in which the experimenters were physically present to provide the gum, this method cannot be employed in the current experiment, as participants will perform the task virtually and are not expected to have gum readily available. Furthermore, it would be difficult to assess compliance with the gum-chewing task via videoconferencing software. Instead, we agreed that a comparable task would be to hold a pen or pencil between the teeth. While this does not involve movement like gum-chewing does, the position does interfere with the ability to manipulate the lips, tongue, and embouchure-related muscles, and we will be able to easily assess via Zoom whether or not the participant is complying with the task.

Using the stimuli described in the Stimuli section above, Experiment 2 comprises a full factorial design crossing the following two two-level factors: transposition (original vs. pitch-shifted), and motor interference (no interference vs. motor interference). Transposition, in this regard, refers to the fact that half of the presented tones have been pitch-shifted as described in the Stimuli section and Table 1 above. Whereas the no interference condition entails no further task in addition to pitch identification, the motor interference condition entails two concurrent tasks which will be performed by the oboists while listening to the stimuli and identifying pitches. Specifically, in this condition, oboists will be asked to hold a pen or pencil horizontally between their teeth and to continuously wiggle the fingers of their left hand.

Based on the experimental design and simulations using the pilot data, it was determined that Experiment 2 will consist of four blocks of 18 trials each (Fig 4). Overall, each participant will judge a total of 36 unique sound files, each presented twice (with and without motor interference) for a total of 72 trials. The 36 unique sound files will consist of 18 different tones presented at their original pitch level as well as the pitch-shifted versions of each of these 18 tones. During two of the four blocks, participants will be instructed to engage in the motor interference task. Participants will not be aware that half of the tones will have been artificially pitch-shifted. The 36 unique stimuli will be randomly distributed across the two blocks of the motor interference condition and across the two blocks of the no motor interference condition, and the 18 stimuli in each block will be presented to each oboist participant in random order. For an illustration of the experimental design, refer to Fig 4. Our experiment will be created and hosted using the Gorilla Experiment Builder (www.gorilla.sc) [42].

The 18 tones tested for each individual oboist will be selected with respect to their performance in Experiment 1; specifically, they will be tested using the 18 tones for which they demonstrated the highest accuracy (with ties determined by random selection). While ideally we would test the full range of the oboe for each participant, given the number of conditions, this would result in an excessively long experiment. By selecting the tones for each participant that yielded the most success in the initial Experiment 1, we aim to increase the likelihood of observing the predicted interference in accuracy. The subset of pitch-shifted tones will be matched in original pitch to the base set of tones selected by means of previous success. This approach avoids a scenario in which the same set of 18 pitch names are correct within each of the four blocks, which, if noticed by the participants, could potentially provide a cue influencing their responses in later blocks. This risk is further minimized through the random distribution of the 36 unique sound stimuli across the two blocks of each type.

After each block, two validity check questions will confirm that participants always listened through headphones and complied with task instructions in terms of holding a pen or pencil between their teeth and moving their left fingers in the motor interference conditions or refraining from doing so in the conditions without motor interference. Following the final block, participants will be asked to complete the same post-experiment questions as described for Experiment 1.

The four blocks of Experiment 2 will be presented in a reverse counterbalanced order for each participant (Fig 4). With two conditions—motor interference (M) and no interference (N)—the two orders (M-N-N-M and N-M-M-N) will be used an equal number of times across the participants in Experiment B.

## Proposed timeline

We anticipate beginning data collection directly after the completion of the peer review process. It is expected that data collection will take four to six months; this length will be in part dependent on the number of participants needed for Experiment 2. Analysis and final write-up will be completed within a further three months.
